# IL-6 Prevents Lung Macrophage Death and Lung Inflammation Injury by Inhibiting GSDME- and GSDMD-Mediated Pyroptosis during Pneumococcal Pneumosepsis

**DOI:** 10.1128/spectrum.02049-21

**Published:** 2022-03-17

**Authors:** Xuemei Gou, Wenchun Xu, Yusi Liu, Yang Peng, Wenlong Xu, Yibing Yin, Xuemei Zhang

**Affiliations:** a Key Laboratory of Diagnostic Medicine Designated by the Ministry of Education, Chongqing Medical University, Chongqing, China; b Department of Laboratory Medicine, Yongchuan Hospital of Chongqing Medical University, Chongqing, China; c Department of Laboratory Medicine, First Hospital of China Medical University, Shenyang, China; University of Georgia

**Keywords:** IL-6, macrophage, inflammation, pyroptosis, pneumococcal pneumosepsis

## Abstract

Streptococcus pneumoniae is a leading bacterial cause of a wide range of infections, and pneumococcal pneumosepsis causes high mortality in hosts infected with antibiotic-resistant strains and those who cannot resolve ongoing inflammation. The factors which influence the development and outcome of pneumosepsis are currently unclear. IL-6 is critical for maintaining immune homeostasis, and we determined that this cytokine is also essential for resisting pneumosepsis, as it inhibits macrophage pyroptosis and pyroptosis-related inflammation injury in the lung. IL-6 affected infection outcomes in mice and exerted a protective role, primarily via macrophages. We further found that IL-6 deficiency led to increased lung macrophage death and aggravated lung inflammation, and that exogenous administration of IL-6 protein could decrease macrophage death and alleviate lung tissue inflammation. IL-6 also protected Streptococcus pneumoniae-induced lung macrophage death and lung inflammation injury by inhibiting gasdermin E (GSDME)- and gasdermin D (GSDMD)-mediated pyroptosis. Together, these data reveal a novel mechanism for the development of pneumosepsis and the critical protective role of IL-6. These findings may assist in the early identification and treatment of pneumococcal pneumosepsis.

**IMPORTANCE** Pneumococcal pneumonia has been a significant cause of morbidity and mortality throughout human history. Failing to control pneumococcal pneumonia and resolve ongoing inflammation in a host can cause sepsis, namely pneumococcal pneumosepsis, and death ensues. Few theories have suggested an optimally therapeutic option for this infectious disease. The interleukin-6 (IL-6, a cytokine featuring pleiotropic activity) theory, proposed here, implies that IL-6 acts as a protector against pneumococcal pneumosepsis. IL-6 prevents lung macrophage death and lung inflammation injury by inhibiting a caspase-3-GSDME-mediated switch from apoptosis to pyroptosis and inhibiting caspase-1-GSDMD-mediated classic pyroptosis during pneumococcal pneumosepsis. Thus, IL-6 is an important determinant for controlling bacterial invasion and a homeostatic coordinator of pneumococcal pneumosepsis. This study clarifies a novel mechanism of occurrence and development of pneumonia and secondary sepsis following a Streptococcus pneumoniae infection. It is important for the early identification and treatment of pneumococcal pneumosepsis.

## INTRODUCTION

Pneumococcal pneumonia is the most common type of bacterial pneumonia, the leading cause of death for children under 5 years old and patients during an influenza epidemic (1, 2), and it is also the main reason why community-intensive pneumonia patients enter the intensive care unit (1, 3). Pneumococcal pneumonia often triggers an intense inflammatory reaction that may be resolved with supportive care, but infections that result in sepsis are often life-threatening (4). The links between pulmonary immunity and systemic disease progression are complex and not completely understood.

Lung macrophages are the predominant immune cells in homeostatic airways (5). Macrophages contribute to the clearance of bacteria through different methods, such as release of antibacterial molecules and macrophage polarization (5–7). There is emerging evidence that lung macrophage death can influence disease progression (7–9). Bacteria have distinct mechanisms to cause monocyte death, some of which are highly proinflammatory, such as pyroptosis and necroptosis. In contrast, some can lead to apoptosis and autophagy, which do not elicit a host inflammation response (10). Apoptosis is tightly linked with Streptococcus pneumoniae (S. pneumoniae) infections (3) and the role of induced apoptosis in the progression of these infections is uncertain. S. pneumoniae-infection-induced pyroptosis (lytic cell death) occurs through caspase-1 activation in murine microglia (10–13). Whether there is a relationship between apoptosis and pyroptosis during pneumococcal pneumosepsis still unknown.

Our previous studies have utilized an influenza A virus and S. pneumoniae coinfection model, and indicated that IL-6 deficiency during an infection results in increased lung cell death (14). It is important to understand whether IL-6-regulated lung cell death is the key mechanism of pneumococcal pneumosepsis. Studies have shown that IL-6 is a direct player in the integrated immune response (15–17), and elevated levels of IL-6 protein and mRNA have been found in the lungs and plasma of patients and in S. pneumoniae-infected mice (18). *IL-6*^−/−^ mice also have impaired defenses against S. pneumoniae infection (17). However, IL-6 levels vary widely depending on the type, severity, and location of the disease, and may result in either protection or exacerbation (19). Therefore, we need to define the direct linkage between IL-6-regulated lung cell death and inflammation and pneumococcal pneumosepsis.

Here, we found that IL-6 promotes bacterial clearance and improves the survival of mice with pneumococcal pneumosepsis via macrophages and inflammation control. IL-6 deficiency in a mouse model led to lung macrophage death and aggravated lung tissue inflammation, and these effects could be relieved by the administration of exogenous IL-6. We further demonstrated that IL-6 contributes to lung macrophage death and lung inflammation injury by inhibiting GSDME- and GSDMD-mediated pyroptosis during pneumococcal pneumosepsis. Thus, elevated IL-6 in mice with pneumococcal pneumosepsis is protective and eliminating IL-6 leads to disease progression. We examined the underlying mechanisms of pneumococcal pneumosepsis and identified a novel mechanism that leads to occurrence and development of pneumonia and secondary sepsis following the S. pneumoniae infection. These data can assist clinicians in the early identification and treatment of pneumosepsis.

## RESULTS

### The outcome of pneumococcal pneumosepsis is significantly affected by IL-6.

**FIG 1:**
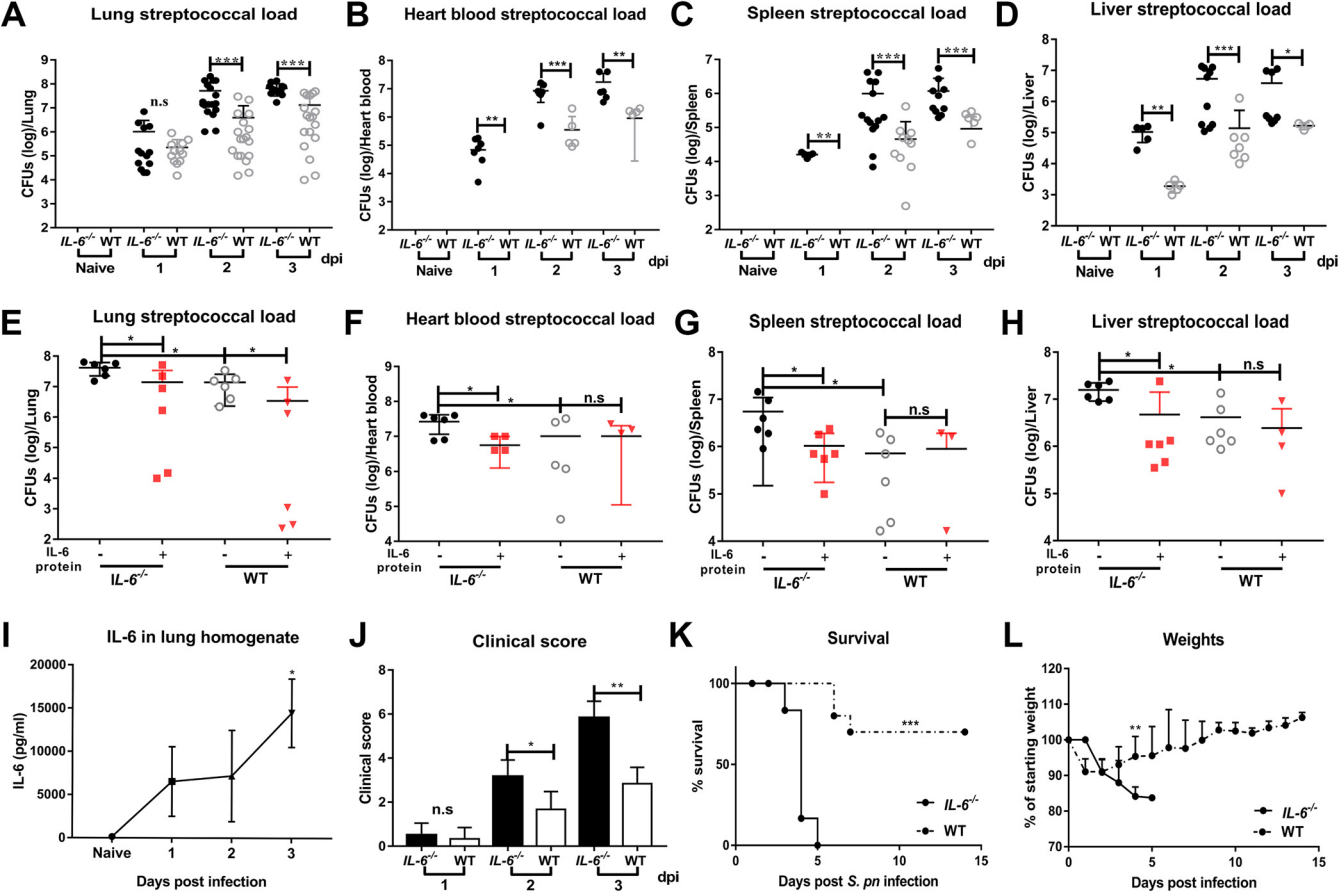
IL-6 is required for bacterial clearance and survival during pneumococcal pneumosepsis. (A to H) Pneumococcal loads of lungs (A, *n* = 12 to 18/group; E, *n* = 6/group), heart blood (B, *n* = 6 to 9/group; F, *n* = 5 to 6/group), spleens (C, *n* = 5 to 16/group; G, *n* = 4 to 6/group), and livers (D, *n* = 5 to 11/group; H, *n* = 5 to 6/group) of *IL-6^−/−^* and WT mice following intranasal infection with 1 × 10^8^ CFU D39 from 1 to 3 dpi (A to D) or following intranasal treatment with exogenous recombinant mouse IL-6 protein (10 μg/30 μL/mouse) during pneumococcal pneumosepsis (E to H). (I to J) Dynamic changes of IL-6 in lungs of WT mice (I, *n* = 5/group, by enzyme-linked immunosorbent assay [ELISA]) and clinical scores (J, *n* = 6/group) of *IL-6^−/−^* and WT mice from 1 to 3 dpi. (K to L) Mortality (K, *n* = 6 to 10/group) and weights (L, *n* = 6/group) following D39 infection, as indicated.

We established a wild-type (WT) mouse model of pneumococcal pneumonia to study the pathogenesis of pneumococcal pneumosepsis. Intranasal instillation of S. pneumoniae D39 at 1 × 10^8^ CFU resulted in CFU levels of 1 × 10^6^ at 24 h postinfection (hpi), indicating effective clearance of the invasive bacteria. However, pneumococcal loads gradually increased at 48 and 72 hpi, indicating a loss of effective bacterial clearance which resulted in bacterial proliferation. Secondary sepsis was determined by measuring pneumococcal loads in heart blood, spleen, and liver of WT mice. The pneumococcal loads in these tissues indicated that bacteria from the lungs diffused after 24 hpi. Interestingly, the *IL-6^−/−^* mice had developed sepsis prior to 24 hpi. This indicated that an IL-6 response was necessary for disease resistance (Fig. 1A to D). Administration of exogenous recombinant mouse IL-6 protein to both *IL-6^−/−^* and WT mice significantly reduced bacterial loads (Fig. 1E to H).

Next, we dynamically monitored IL-6 levels. IL-6 levels in healthy mice were initially low, and gradually increased at different times postinfection (Fig. 1I). Infected *IL-6^−/−^* and WT mice did not differ in clinical signs at 24 hpi, but these became significantly more obvious in *IL-6^−/−^* mice as time progressed (Fig. 1J). Consistent with these observations, most (70%) WT mice recovered from the infection and suffered only mild weight loss as expected, while all of the *IL-6^−/−^* mice died with substantial weight loss (Fig. 1K and L). These results indicated that IL-6 plays an important role in eliminating invasive S. pneumoniae and determining infection outcomes.

### IL-6 affects the outcome of mice with pneumococcal pneumosepsis mainly through its effect on macrophages.

Approximately 95% of airspace leukocytes are macrophages, and these can be activated by IL-6 (5, 8). We therefore explored whether the outcomes of pneumococcal pneumosepsis were IL-6-dependent through its effect on macrophages. We administered WT bone marrow-derived macrophages (BMDM) by intranasal instillation and determined bacterial loads in bronchoalveolar lavage fluid (BALF), lung, spleen, and liver. BMDM in the lungs of both *IL-6^−/−^* and WT mice significantly promoted bacterial clearance (see Fig. S1A–D in the supplemental material). Histopathology of lung tissue and total protein in BALF were used as indices to quantify inflammatory exudates in the lungs. The results indicated that sufficient BMDM were present to significantly reduce lung inflammation in *IL-6^−/−^* and WT mice. In addition, there was more obvious lung inflammation injury in *IL-6^−/−^* mice compared to WT mice, and there was no difference between *IL-6^−/−^* mice with BMDM and WT mice (with high IL-6) (Fig. S1E–G). We also found the infected WT mice had a defect in bacterial clearance when given *IL-6^−/−^* BMDM by intranasal instillation (Fig. S1H). This result further confirmed the important effect of IL-6 on macrophages. These results suggested that IL-6 exerts antibacterial and anti-inflammatory effects in a macrophage-dependent manner.

**FIG 2:**
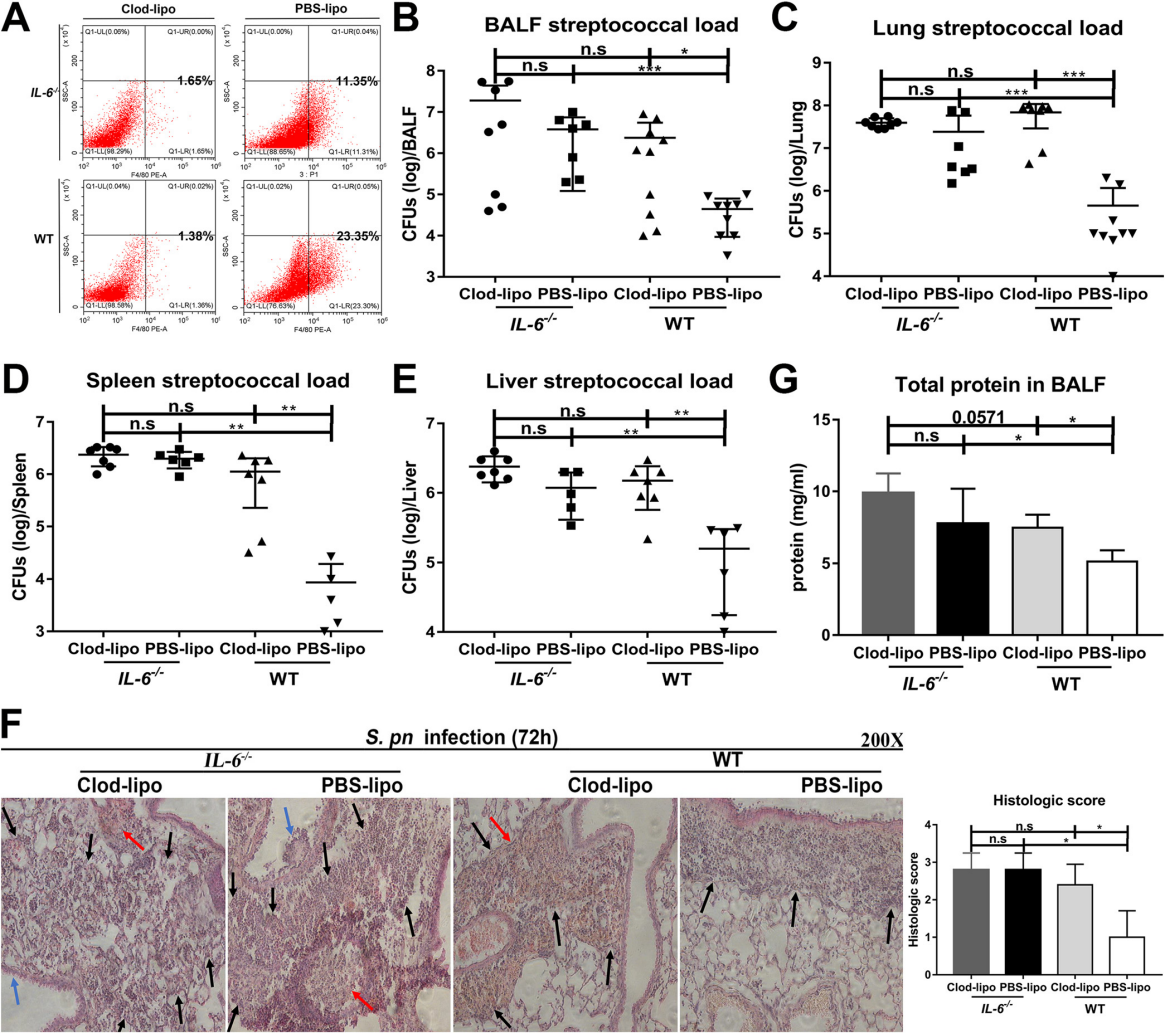
Depletion of lung macrophages counteracts the antibacterial and anti-inflammatory effects of IL-6. Lung macrophages were depleted by intraperitoneal administration of clodronate-liposomes. (A) F4/80^+^ macrophages in BALF after depletion (*n* = 6 to 7/group). Pneumococcal loads for (B) BALF (*n* = 7 to 10/group), (C) lungs (*n* = 7 to 9/group), (D) spleens (*n* = 5 to 7/group), and (E) livers (*n* = 5 to 7/group). (F) Hematoxylin and eosin (H&E) staining of lung tissue sections (*n* = 5/group) and (G) total protein in BALF (*n* = 4 to 5/group). Black arrow, inflammatory cell infiltration; blue arrow, epithelial cell shedding; red arrow, bleeding.

We next depleted lung macrophages in an attempt to verify that the effects of IL-6 were due to its effect on macrophages. Lung macrophage depletion was verified by flow cytometry (Fig. 2A). Depletion in WT mice resulted in increased bacterial loads in BALF, lung, spleen, and liver, suggesting that macrophages played important roles in bacterial clearance in the presence of IL-6. In contrast, regardless of depletion of lung macrophages in *IL-6^−/−^* mice, there was no effect on bacterial clearance, indicating that IL-6 was important in host defense against infection. Additionally, there was no difference in bacterial clearance between *IL-6^−/−^* and WT mice after macrophage depletion (Fig. 2B to E). Collectively, these results implied that IL-6 performs its role via macrophages.

Depletion of lung macrophages significantly increased lung pathology in WT mice, and these effects included edema, hyperemia and congestion, inflammatory cell recruitment, intra-alveolar hemorrhage and debris, and destruction of alveolar structure. In contrast, lung injury in *IL-6^−/−^* mice was independent of macrophage depletion; macrophage depletion significantly increased total protein in the BALF of WT mice, but had no effect on this parameter in *IL-6^−/−^* mice. Similarly, there was no difference in histopathology and inflammation exudation between *IL-6^−/−^* and WT mice after the depletion of macrophages (Fig. 2F and G). This provided additional support for a role of IL-6 that is exerted on macrophages.

### IL-6 deficiency is not conducive to lung macrophage presence and control of lung inflammation during pneumococcal pneumosepsis.

**FIG 3:**
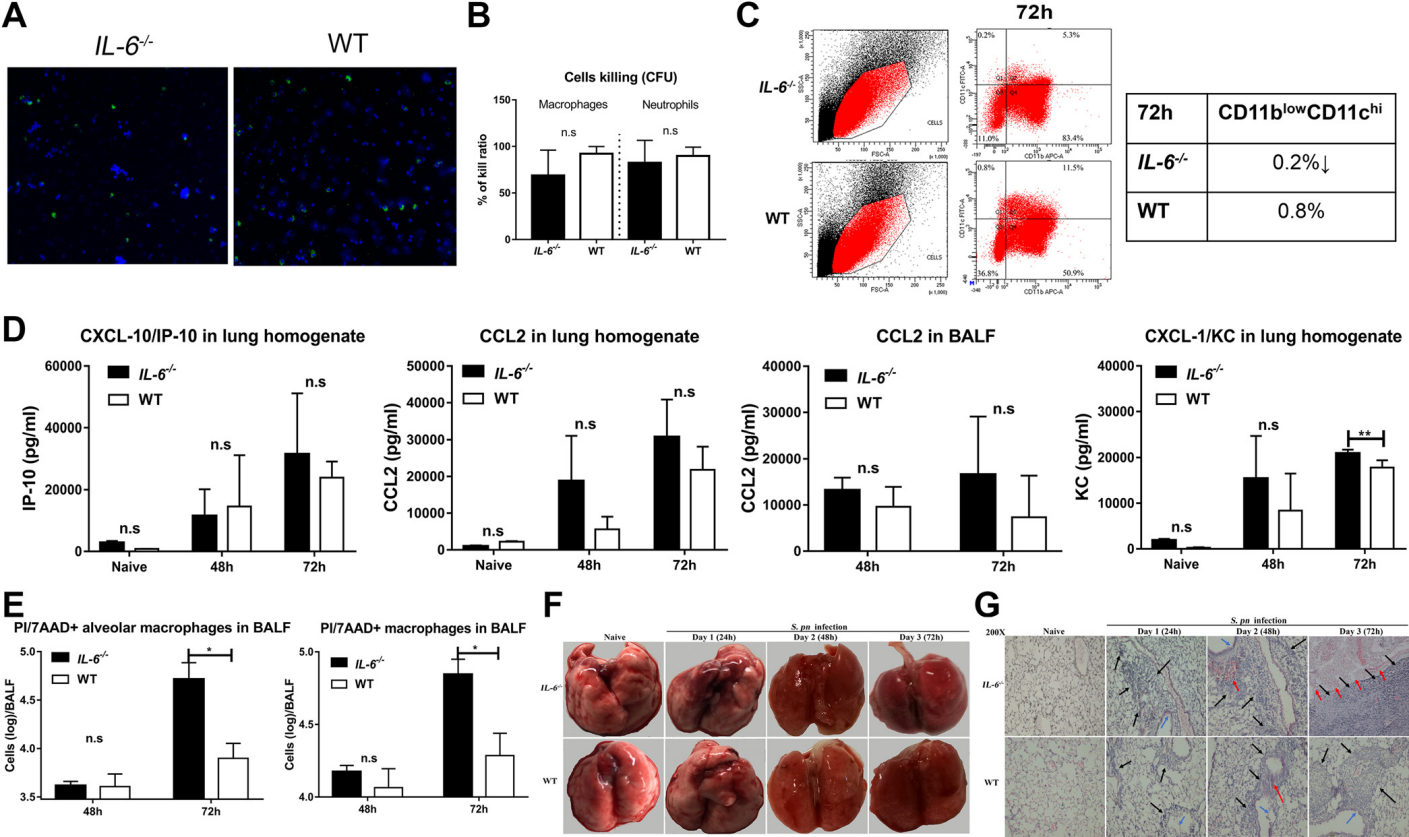
IL-6 is crucial for lung macrophage survival during pneumococcal pneumosepsis. (A) *In vivo* phagocytosis assays of mice (*n* = 3/group). (B) *In vitro* CFU-based S. pneumoniae killing analysis of peritoneal macrophages and neutrophils (*n* = 4 to 9/group). (C) Percentage of CD11b^low^CD11c^hi^ cells in BALF of mice during pneumococcal pneumosepsis (*n* = 3/group). (D) CXCL-10, CCL2, and CXCL-1 levels in lungs of mice at 48 and 72 hpi were measured by ELISA, (*n* = 4 to 6/group). (E) Propidium iodide/7-aminoactinomycin D (7-AAD^+^)-staining of alveolar and resident lung macrophages of *IL-6^−/−^* and WT mice at 48 and 72 hpi (*n* = 4 to 5/group). (F to G) Representative photomicrographs of lung tissue histopathology (*n* = 3/group) and H&E-stained tissues (*n* = 3/group) of *IL-6^−/−^* and WT mice from 1 to 3 dpi. Black arrow, inflammatory cell infiltration; blue arrow, epithelial cell shedding; red arrow, bleeding.

We measured alterations in lung macrophages to determine whether these cells were regulated by IL-6. Unexpectedly, *in vivo* and *in vitro* phagocytosis assays indicated that IL-6 deficiency did not significantly alter major phagocyte functions (Fig. 3A and B). However, lung macrophage numbers and percentages in *IL-6^−/−^* mice were significantly lower than those in WT mice during pneumococcal pneumosepsis (Fig. S2). We further verified these changes in alveolar macrophages using CD11b^low^CD11c^hi^ markers (20). We found that the percentage of resident lung macrophages in *IL-6^−/−^* mice was lower than that in WT mice (Fig. 3C). Previous studies have shown that a decreased ability to recruit macrophages to infection sites can result in local expansive bacterial growth (21). Therefore, we determined whether the decrease in lung macrophages was due to decreased levels of cognate chemokines. IL-6 deficiency did not alter IP-10 and CCL2 levels in *IL-6^−/−^* compared to those in WT mice in lung tissue and BALF (Fig. 3D). However, the absence of IL-6 resulted in increased KC, CXCL-2, and CXCL-5, and was accompanied by increased neutrophil numbers (Fig. 3D and Fig. S2, S3). Much of this decrease in macrophage numbers could be attributed to macrophage death. *IL-6^−/−^* mice with pneumococcal pneumosepsis had elevated levels of propidium iodide/7-aminoactinomycin D (PI/7-AAD^+^) staining lung macrophages in the BALF, indicating a high number of dead cells (Fig. 3E).

Lung macrophage death and lung tissue inflammation form a positive feedback cycle that ultimately augments injury and disease development (8, 22). We therefore examined lung inflammation injury in *IL-6^−/−^* and WT mice during pneumococcal pneumosepsis. Edema, hyperemia, and congestion were obvious in both groups, suggesting a disorder in immunity and coagulation, although *IL-6^−/−^* mice were affected to a greater extent (Fig. 3F). The lung tissues of the *IL-6^−/−^* mice sustained greater levels of inflammation and injury, including more inflammatory cell recruitment, intra-alveolar hemorrhage and debris, and destruction of alveolar structure (Fig. 3G). These data indicated that the absence of IL-6 resulted in decreased levels of lung macrophages and the loss of immune control, which provided conditions for secondary sepsis after pneumonia.

### IL-6 regulates *S. pneumoniae*-induced macrophage death through GSDME- and GSDMD-mediated pyroptosis.

**FIG 4:**
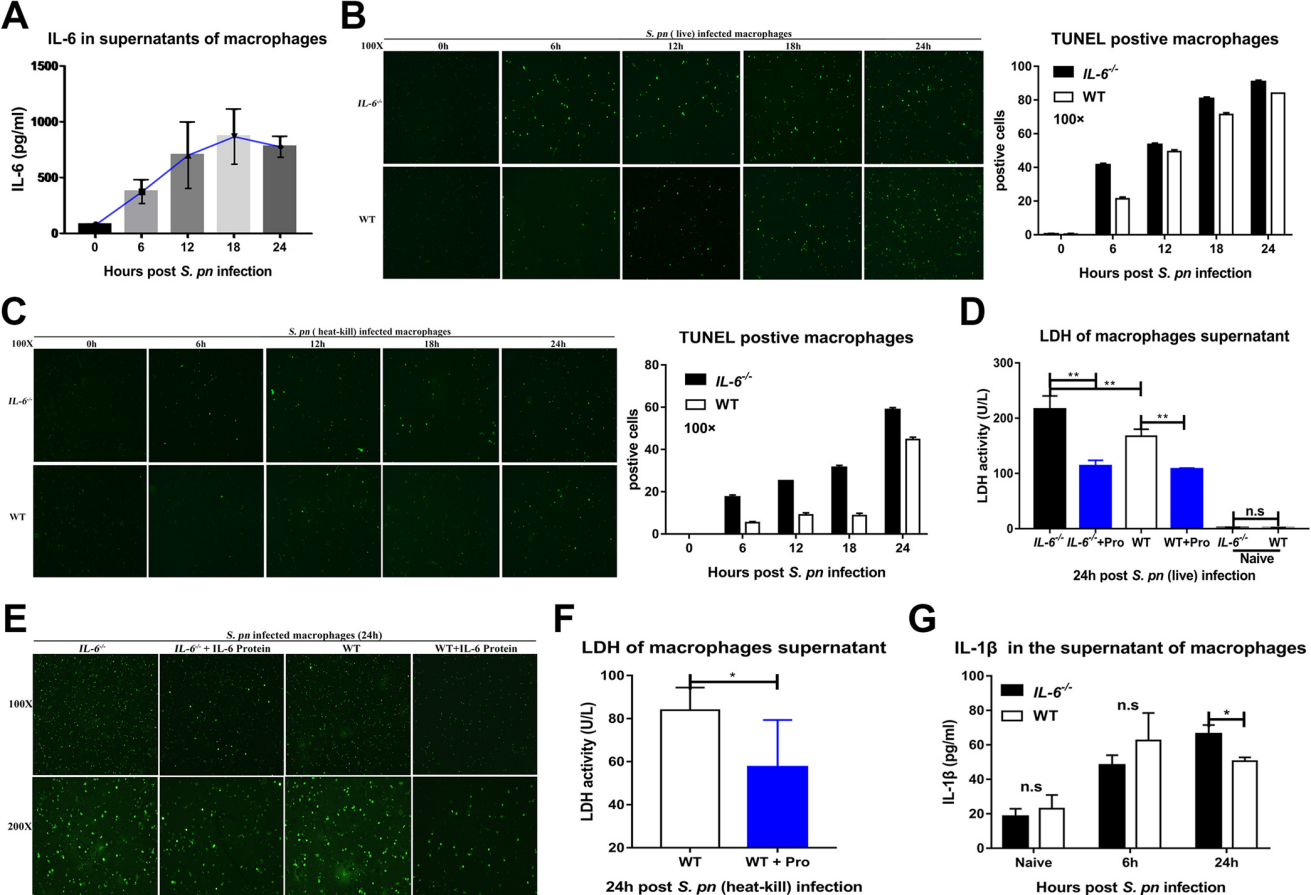
IL-6 alters macrophage survival *in vitro* during S. pneumoniae infection. (A) Dynamic changes of IL-6 in the supernatants of WT peritoneal macrophages at the indicated times following S. pneumoniae infection, as measured by ELISA (*n* = 4 to 6/group). Peritoneal macrophage death was assessed by *in situ* cell death detection kit *in vitro* after infection with (B) live (*n* = 3/group) and (C) heat-killed (*n* = 3/group) S. pneumoniae. Peritoneal macrophage death was assessed by LDH release (D, *n* = 6/group; F, *n* = 5/group) and (E) photomicrographs of cell death (*n* = 3/group) in the presence and absence of exogenous recombinant mouse IL-6 protein as indicated. (G) IL-1β levels in the supernatants of S. pneumoniae-infected macrophages at the indicated times (*n* = 4/group).

To further clarify the specific regulatory features exerted by IL-6 on macrophage death, we collected peritoneal macrophages from mice and infected them with S. pneumoniae *in vitro*. IL-6 levels gradually increased and peaked at 18 to 24h (Fig. 4A). Macrophages which lacked IL-6 were killed early in infection (Fig. 4B). Interestingly, similar results were obtained when using heat-inactivated S. pneumoniae, although the overall number of cells that died was reduced (Fig. 4C). Macrophages lacking IL-6 also displayed high levels of lactate dehydrogenase (LDH) in the culture supernatants, indicating cell death, whereas the addition of IL-6 protein significantly reduced LDH in infected *IL-6^−/−^* and WT macrophages (Fig. 4D). Similarly, a commercial kit that measures *in situ* cell death indicated that exogenous IL-6 protein significantly protected both *IL-6^−/−^* and WT macrophages from death at 24 hpi (Fig. 4E). Supporting this, IL-6 protein also significantly reduced LDH release from macrophages infected with heat-inactivated S. pneumoniae (Fig. 4F).

**FIG 5:**
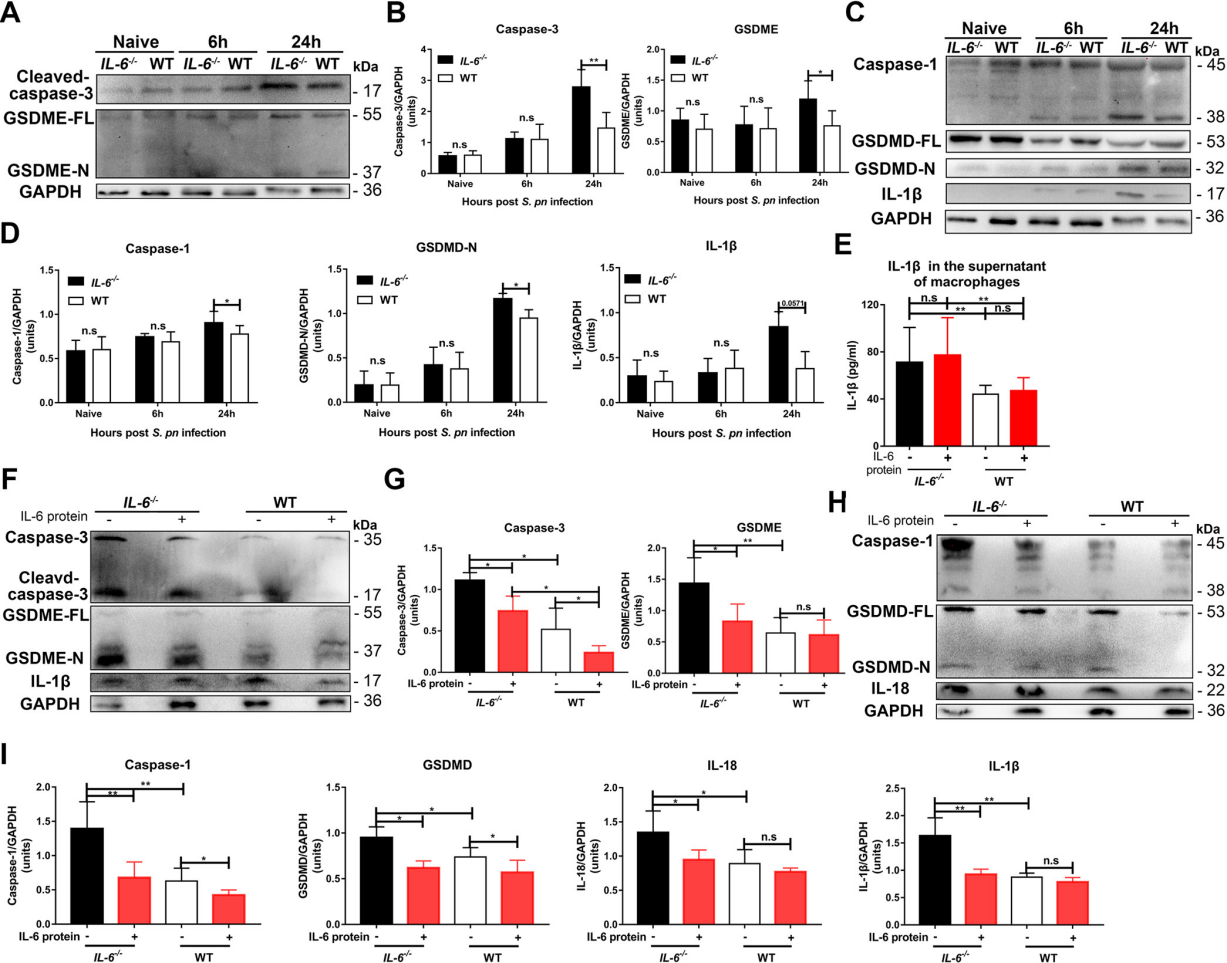
IL-6 regulates macrophage death through GSDME- and GSDMD-mediated pyroptosis *in vitro* during S. pneumoniae infection. (A and B) Caspase-3 (*n* = 5/group) and GSDME (*n* = 5/group) and (C and D) caspase-1 (*n* = 3 to 8/group), GSDMD (*n* = 5/group) and IL-1β (*n* = 5/group) in cell lysates of S. pneumoniae*-*infected peritoneal macrophages at the indicated times. (E) IL-1β levels in the supernatants (*n* = 10/group) of S. pneumoniae infected peritoneal macrophages from *IL-6^−/−^* and WT mice with or without IL-6 protein treatment as indicated. (F and G) Caspase-3 (*n* = 4/group), GSDME (*n* = 6/group) and IL-1β (*n* = 5/group) and (H and I) caspase-1 (*n* = 5/group), GSDMD (*n* = 4 to 5/group) and IL-18 (*n* = 4/group) in S. pneumoniae infected peritoneal macrophages with or without exogenous recombinant mouse IL-6 protein (see above) at 24 hpi.

Apoptosis is a recognized feature of S. pneumoniae infections (3), and this bacterium can also induce pyroptosis through caspase-1 activation (11). It also has been reported that caspase-3-activated GSDME causes a switch from caspase-3-mediated apoptosis to pyroptosis (12, 23). Excessive pyroptosis causes various inflammatory diseases, such as sepsis (12, 23). Our studies found that IL-6 could alter the apoptosis of lung cells in BALF and lung tissues (Fig. S4A–D). Infected *IL-6^−/−^* and WT macrophages both released IL-1β, with the maximum release at 24 hpi (Fig. 4G). This cytokine is associated with pyroptosis, but not with apoptosis (10, 12, 23). We also found that caspase-3 and GSDME expression in *IL-6^−/−^* macrophages were high at 24 hpi (Fig. 5A and B). Furthermore, caspase-1 and GSDMD levels in the *IL-6^−/−^* macrophages exceeded those in the WT macrophages (Fig. 5C and D). Accordingly, IL-1β and IL-18 levels in the cell lysates and culture supernatants of *IL-6^−/−^* macrophages were significantly increased at 24 hpi (Fig. 4G, Fig. 5C to E). The addition of exogenous IL-6 protein to these two cultured cells was able to decrease GSDME- and GSDMD-mediated pyroptosis (Fig. 5F to I).

**FIG 6:**
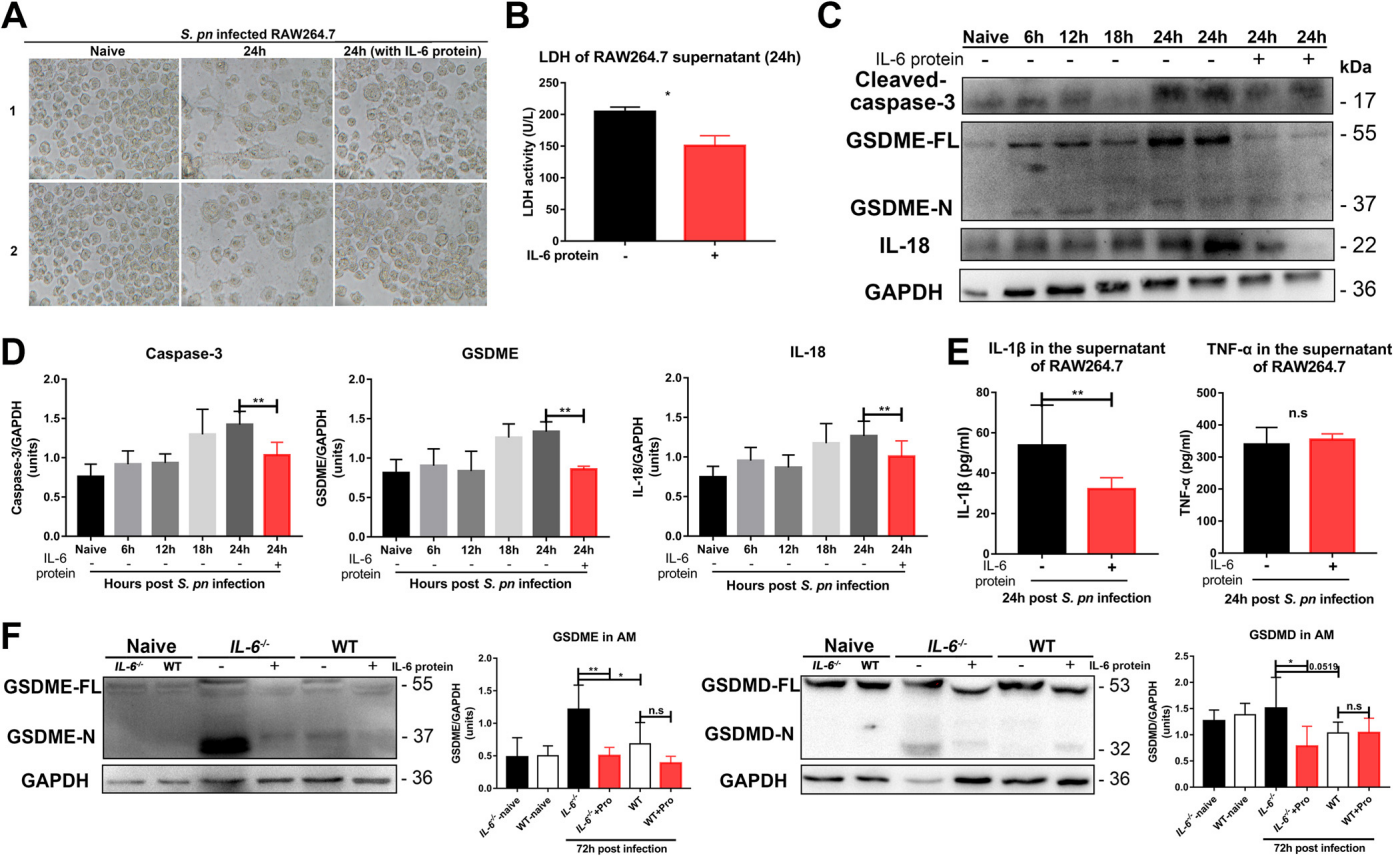
IL-6 regulates cell death in cultured RAW264.7 cells and alveolar macrophages through GSDME- and GSDMD-mediated pyroptosis. (A) Photomicrographs of RAW264.7 cells in the presence and absence of S. pneumoniae 24 hpi with or without recombinant mouse IL-6 protein (20 ng/mL) as indicated (*n* = 3/group). (B) RAW264.7 cell death was assessed by lactate dehydrogenase (LDH) release (*n* = 4/group). (C to D) Caspase-3 (*n* = 3 to 5/group), GSDME (*n* = 3 to 6/group), and IL-18 (*n* = 5 to 12/group) in lysates of S. pneumoniae-infected RAW264.7 cells at the indicated times. (E) IL-1β (*n* = 6/group) and TNF-α (*n* = 5/group) in the supernatants of S. pneumoniae-infected RAW264.7 cells at 24 hpi, with or without IL-6 protein, as above. (F) GSDME (*n* = 3 to 8/group) and GSDMD (*n* = 3 to 6/group) in the cell lysates of S. pneumoniae-infected alveolar macrophages at the indicated times.

As additional verification, we used the macrophage cell line RAW264.7 to determine whether these results could be independently duplicated. S. pneumoniae-infected RAW264.7 cells displayed morphologies, sizes, and cell numbers that were distinct from those of uninfected cells. We noted obvious cell shrinkage, suggesting an apoptotic process, but also swelling, suggesting a pyroptotic process. Interestingly, the addition of exogenous IL-6 protein prevented these gross morphological alterations (Fig. 6A and B). We also found evidence for the presence of GSDME-mediated pyroptosis signaling pathways in S. pneumoniae-infected cells, and the addition of exogenous IL-6 protein could significantly prevent this occurrence (Fig. 6C and D). Exogenous IL-6 protein also inhibited secretion of IL-1β, but not secretion of tumor necrosis factor α (TNF-α), indicating a pyroptosis-specific effect (Fig. 6E). RAW264.7 cells expressed endogenous NLRP3, but not ASC (24), and thus possesses a defect in the activation of caspase-1 signaling. This provided indirect evidence for a link between IL-6 and pyroptosis. Finally, we examined alveolar macrophages in the BALF of *IL-6^−/−^* and WT mice. We obtained consistent results with the peritoneal macrophages and the RAW264.7 cells (Fig. 6F).

Taken together, our results indicated that S. pneumoniae-induced pyroptosis was activated through a GSDME-mediated switch from apoptosis to classical GSDMD-mediated pyroptosis. IL-6 deficiency led to an exacerbation of this switch, and the administration of exogenous IL-6 therefore blocked it.

### IL-6 prevents GSDME- and GSDMD-mediated lung inflammation injury during pneumococcal pneumosepsis.

**FIG 7:**
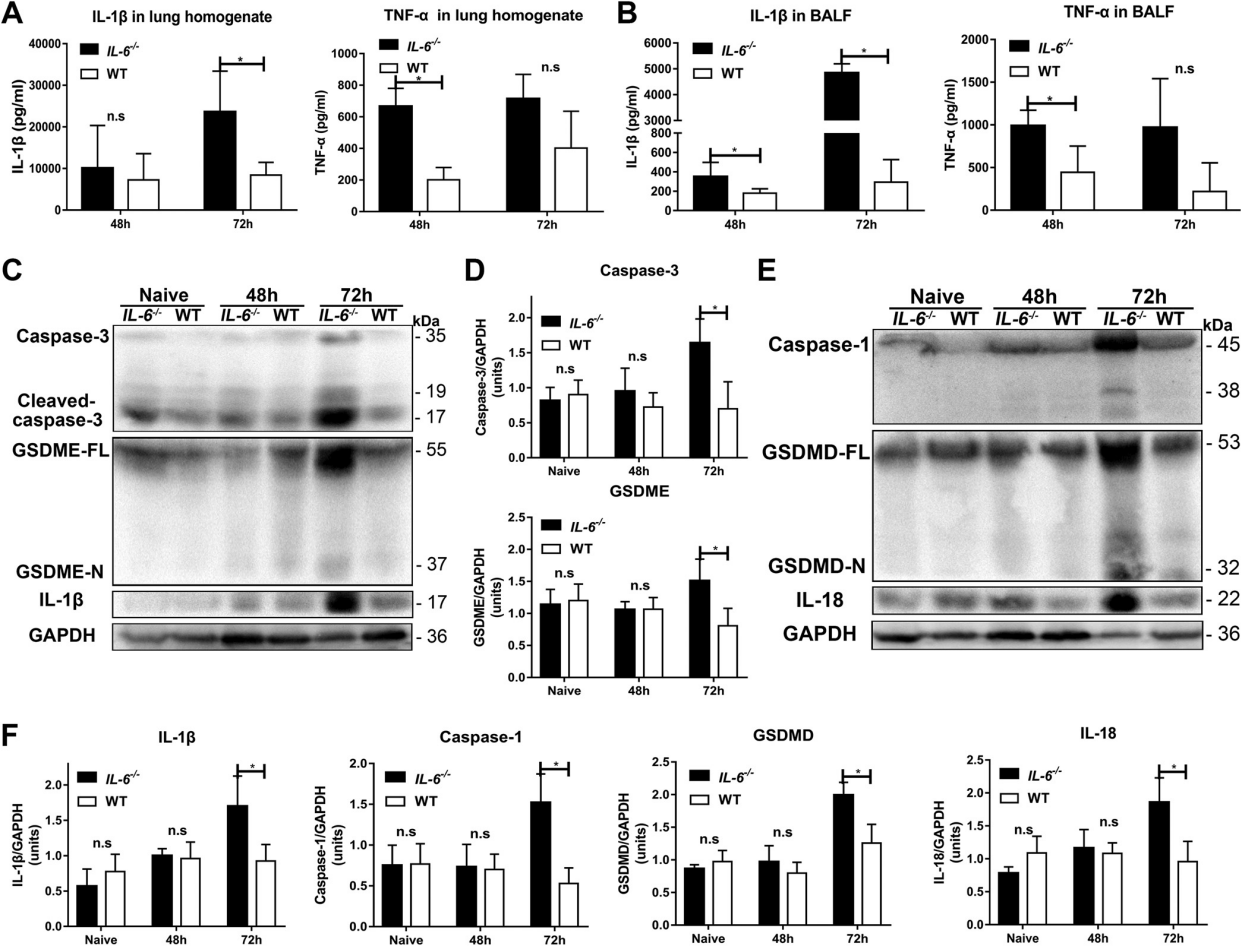
IL-6 deficiency causes aggravated lung injury due to increased GSDME- and GSDMD-mediated pyroptosis *in vivo*. IL-1β and TNF-α in (A) lung homogenates (*n* = 5/group) and (B) BALF (*n* = 5/group) at 48 and 72 hpi. (C and D) Protein levels of caspase-3 (*n* = 4/group), GSDME (*n* = 4/group), and IL-1β (*n* = 4/group); and (E and F) caspase-1 (*n* = 4/group), GSDMD (*n* = 4/group), and IL-18 (*n* = 4/group) in lungs of mice at 48 and 72 hpi, as indicated.

Lung macrophage death and lung tissue inflammation reciprocally affect each other, ultimately leading to the development of disease (8, 22, 25). We therefore examined whether IL-6 affects lung inflammation injury during pneumococcal pneumosepsis in a mouse model. IL-1β levels in the lung homogenates and BALF of *IL-6^−/−^* mice at 72 hpi were greater than those in their WT counterparts, although TNF-α levels were similar (Fig. 7A and B). These results were consistent with the presence of pyroptotic cells, which secrete large amounts of IL-1β (10, 12, 23). Pyroptosis-signaling pathways in the lung tissues of *IL-6^−/−^* mice showed increased GSDME- and GSDMD-mediated pyroptosis at 72 hpi. These results demonstrated that IL-6 deficiency aggravated lung tissue inflammation in the pneumococcal pneumosepsis model via GSDME- and GSDMD-mediated pyroptosis (Fig. 7C to F).

**FIG 8:**
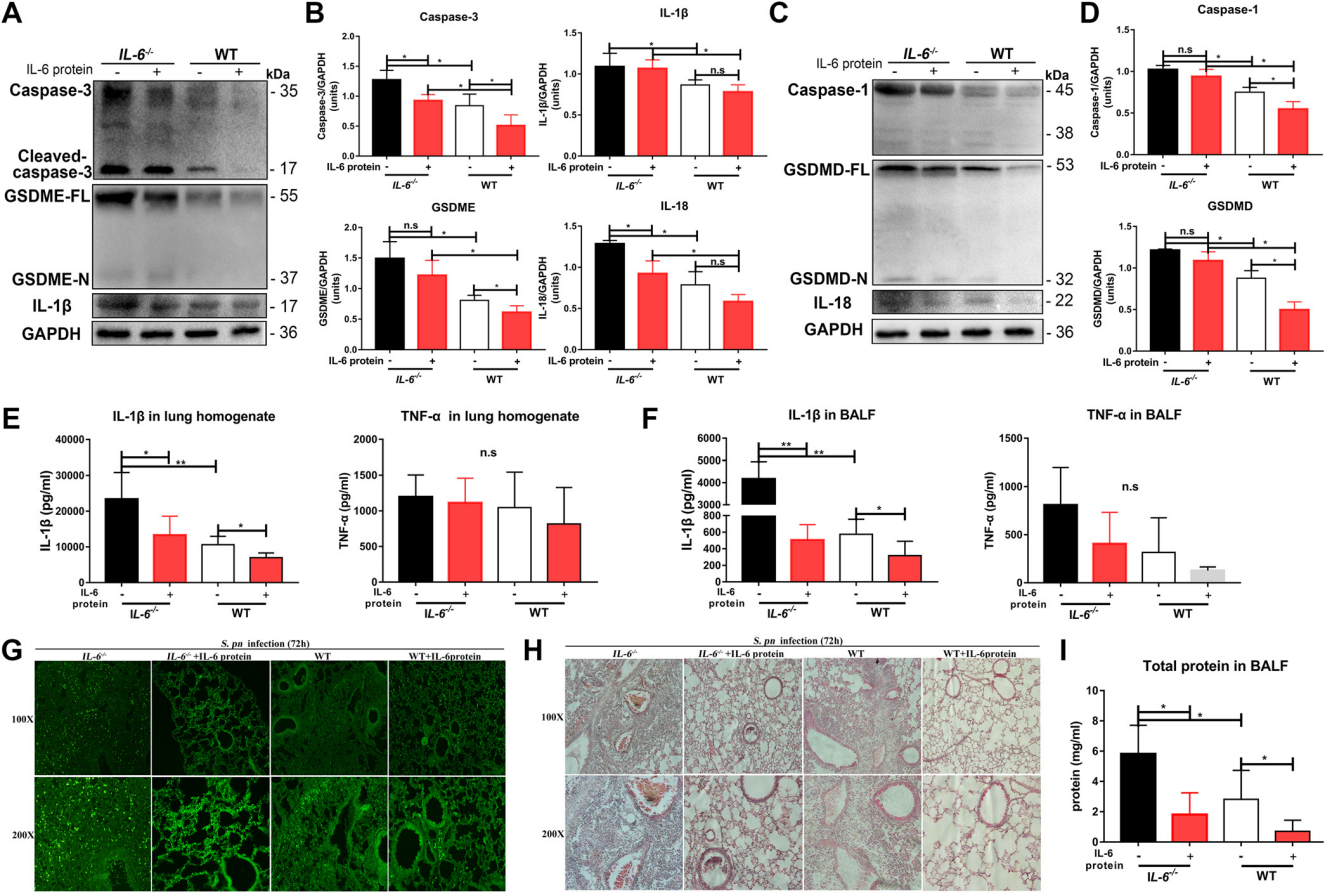
IL-6 prevents GSDME- and GSDMD-mediated lung inflammation injury during pneumococcal pneumosepsis. (A and B) Caspase-3 (*n* = 4/group), GSDME (*n* = 4/group), IL-1β (*n* = 4/group), and (C and D) caspase-1 (*n* = 4/group), GSDMD (*n* = 4/group), and IL-18 (*n* = 4/group) in lungs of mice with or without IL-6 protein treatment during pneumococcal pneumosepsis, as indicated. IL-1β and TNF-α in (E) lung homogenates (*n* = 6/group) and (F) BALF (*n* = 5 to 6/group) of mice with or without IL-6 protein treatment during pneumococcal pneumosepsis, as indicated. (G to I) Lung inflammation injury in mice with or without IL-6 protein treatment was assessed *in situ* using (G) a commercial cell death detection kit (*n* = 3/group), (H) H&E staining (*n* = 3/group), and (I) total protein in BALF (*n* = 4 to 7/group) during pneumococcal pneumosepsis.

We next assessed mRNA levels of these proteins to determine whether they were affected by IL-6 at the transcriptional level. Interestingly, we found that in both *IL-6^−/−^* and WT mice, the mRNA levels we measured were similar, with the exception of *Il1β* levels, which were higher in the *IL-6^−/−^* mice (Fig. S5). These results, therefore, indicated that these proteins are primary regulated by IL-6 *via* a post-transcriptional process. The addition of exogenous IL-6 protein to both *IL-6^−/−^* and WT infected mice significantly prevented lung tissue damage from GSDME- and GSDMD-mediated pyroptosis (Fig. 8A to D). Exogenous IL-6 protein also significantly inhibited IL-1β secretion, but had no effect on TNF-α secretion, during pneumococcal pneumosepsis (Fig. 8E and F). Consistent with these results, *in situ* cell death assays and hematoxylin and eosin (H&E) staining in lung tissues indicated that exogenous IL-6 protein significantly prevented lung cell death in both *IL-6^−/−^* and WT mice during pneumococcal pneumosepsis (Fig. 8G). Exogenous IL-6 protein also significantly reduced inflammatory cell recruitment and intra-alveolar hemorrhage and debris, and preserved the overall alveolar structure and lung inflammatory exudates in both *IL-6^−/−^* and WT mice (Fig. 8H and I).

Collectively, these results demonstrated that IL-6 deficiency aggravated lung tissue inflammation in the pneumococcal pneumosepsis model, and that this process could be alleviated by the administration of exogenous IL-6 protein. This process was regulated by the inhibition of GSDME- and GSDMD-mediated pyroptosis during pneumococcal pneumosepsis.

## DISCUSSION

**FIG 9:**
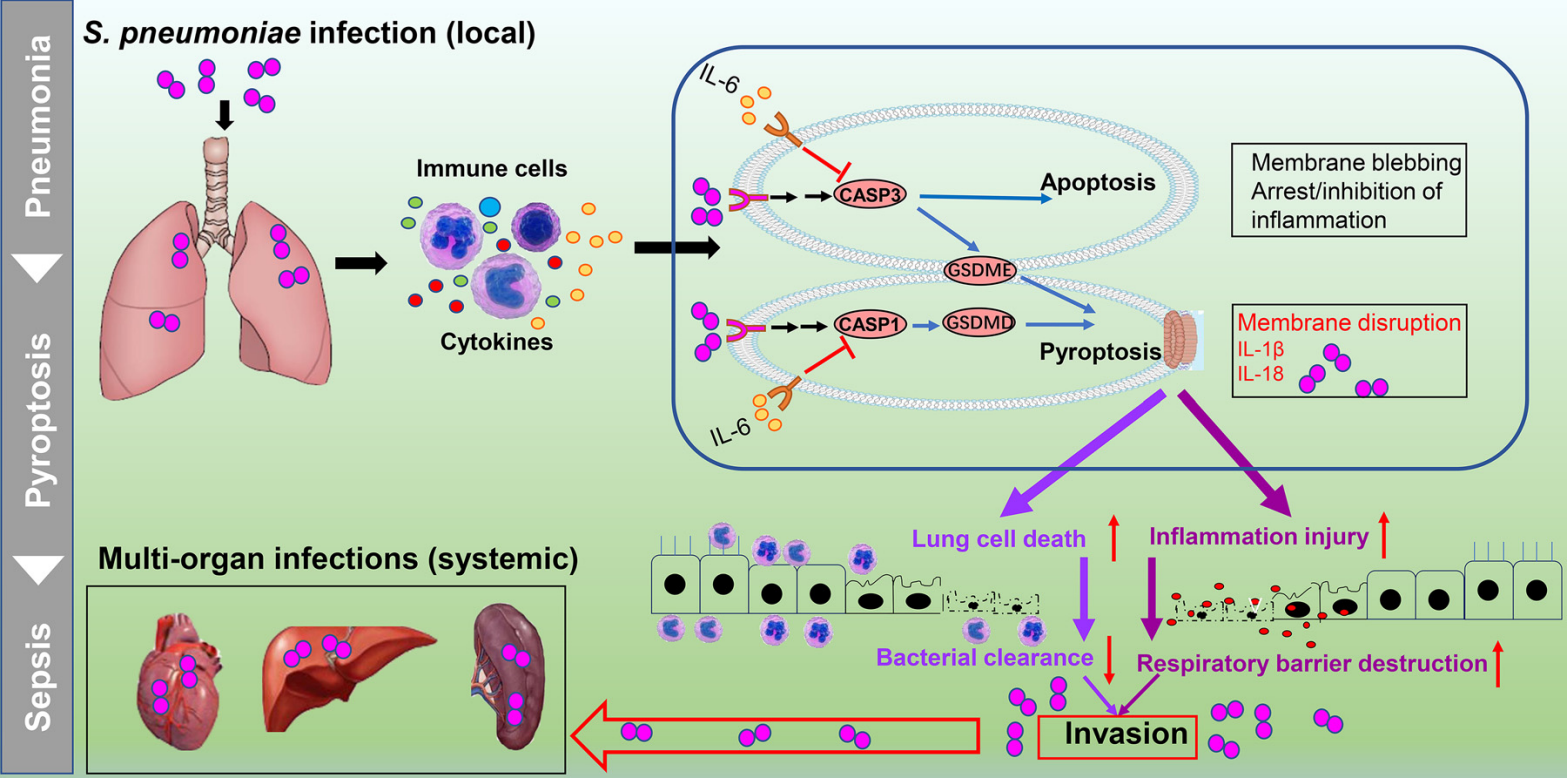
IL-6 prevents lung macrophage death and lung inflammation injury by inhibiting GSDME- and GSDMD-mediated pyroptosis during pneumococcal pneumosepsis. IL-6 prevents S. pneumoniae-induced lung macrophage death and lung inflammation injury by inhibiting GSDME- and GSDMD-mediated pyroptosis.

IL-6 levels increase rapidly when homeostasis is disrupted, and IL-6 is a marker for numerous disease states (19). However, IL-6 levels vary widely depending on the type, severity, and location of disease, and may be the result of either disease protection or exacerbation. IL-6 serves as an anti-inflammatory braking system for macrophages (26), and inflammation-induced IL-6 can contribute to antibacterial protection against S. pneumoniae infection (27). However, a causal relationship is uncertain because of insufficient studies. In this work, we found that IL-6 decreases S. pneumoniae-induced lung macrophage death and alleviates lung inflammation injury during pneumococcal pneumosepsis (Fig. 9). A number of studies have indicated that the pyroptotic response results in the release of antimicrobial molecules and clearance of the infection; in contrast, aberrant cytosolic components could trigger inappropriate pyroptosis, resulting in multiple adverse attacks during infection (13). Whether IL-6 regulation of pyroptosis is beneficial or harmful has been undefined. To address this issue, we studied the effects of IL-6 on pyroptosis during pneumococcal pneumosepsis. Our *in vitro* and *in vivo* results provided evidence for a harmful contribution of pyroptosis during resistance to S. pneumoniae infection. IL-6 treatment significantly prevented this harmful pyroptosis.

Pyroptosis is a gasdermin-dependent form of necrotic cell death (23) where GSDMD is cleaved by caspases 1/11, promoting its oligomerization to form large pores in the plasma membrane and ultimately leading to cell death (10, 23). IL-6 can promote caspase-1 activation and subsequent IL-1β induction (28). Studies have also reported that IL-6 can be activated through a caspase-1-dependent mechanism (29). Considering the reciprocal regulation between IL-6 and caspase-1, we examined whether IL-6 regulates pyroptosis through the caspase-1-GSDMD canonical pathway. Our study demonstrated that IL-6 negatively regulated caspase-1-GSDMD-IL-1β signaling, resulting in decreased lung macrophage death and alleviated lung inflammation injury. GSDME also has been reported to play a role in pyroptosis, where GSDME is cleaved by caspase-3 and switches caspase-3-mediated apoptosis to pyroptosis (12, 23). IL-6 can prevent apoptosis by blocking caspase-3 activation (30) and can also promote apoptosis under specific circumstances (31). IL-6 might regulate the caspase-3-GSDME-mediated switch from apoptosis to pyroptosis; in support of this, we found that IL-6 inhibited caspase-3-GSDME-IL-1β signaling. Thus, we demonstrated that IL-6 regulated pyroptosis through GSDME- and GSDMD-mediated signaling.

Following nasal instillation of recombinant mouse IL-6 protein, we found a protective role of IL-6 in pneumococcal pneumosepsis. However, in some instances, the administration of IL-6 in *IL-6^−/−^* mice did not result in significant changes which could be attributed to an IL-6 dose effect; an increased dose may result in rescue (see Fig. 9B and D). Experiments utilizing exogenously administered IL-6 in inflammatory circumstances have suggested that it provides a beneficial effect (32, 33). Both systemically and locally administered IL-6 decrease neutrophil extravasation, inhibit secretion of inflammatory cytokines such as TNF-α, decrease tissue permeability, and improve animal survival (33). Our future experiments will focus on more clearly defining this role of IL-6.

In summary, this study demonstrated a novel mechanism by which IL-6 prevents lung macrophage depletion and lung injury through the inhibition of GSDME- and GSDMD-mediated pyroptosis. Lung macrophages, in turn, play an important role in defense against early bacterial infection and late systemic infection in the lungs. These findings provide a rationale for immunotherapy of pneumococcal pneumosepsis through modulation of the effect of IL-6 on GSDME- and GSDMD-mediated pyroptosis. This novel mechanism provides us with new information for generating therapeutic strategies against severe infectious diseases.

## MATERIALS AND METHODS

### Animals and murine model of pneumococcal pneumosepsis.

C57/BL/6J mice, 7 to 8 weeks old, of both genders were obtained from Beijing Huafukang Bioscience (Beijing, China) and bred at Chongqing Medical University. *IL-6^−/−^* mice were purchased from the Jackson Laboratory (Bar Harbor, ME). All animal studies were reviewed and approved by the Animal Ethics Committee of Chongqing Medical University. Mice were anesthetized by 1.5% pentobarbital sodium solution.

Pneumococcal pneumosepsis was induced in anesthetized mice intranasally (i.n.) with 1 × 10^8^ CFU S. pneumoniae D39 suspended in 30 μL phosphate-buffered saline (PBS). This process mimicked the natural route of pneumococcal infection (1, 17, 31). Bacterial burdens were measured by euthanizing infected mice at the indicated time points and plating serial 10-fold dilutions of each sample onto blood agar plates. Clinical scores were determined as previously described (34).

### Cell culture.

BMDM and neutrophils were isolated from mouse bone marrow as previously described (14). BMDM were cultured with Dulbecco’s modified Eagle medium (Gibco, USA) supplemented with M-CSF at 10 ng/mL (PeproTech, Inc., USA). Neutrophils were purified using microbeads (Miltenyi Biotec, Germany). Peritoneal macrophages were collected through peritoneal lavage, as previously described (14). Murine RAW264.7 cells were purchased from Shanghai Zhong Qiao Xin Zhou Biotechnology (Shanghai, China).

### Lung macrophage transplantation experiment with BMDM.

Viable WT BMDM (3 × 10^5^) were transferred to WT and *IL-6^−/−^* mice and *IL-6^−/−^* BMDM (3 × 10^5^) were transferred to WT mice intranasally simultaneously with S. pneumoniae (see above). All mice were euthanized for analysis at 72 hpi.

### Lung macrophage depletion.

Lung macrophages were depleted by peritoneal injection of 100 μL clodronate liposomes (Liposoma BV, Netherlands) and intranasal instillation of 50 μL clodronate liposomes at 3 and 1 days prior to infection.

### Histology of lung tissue.

Lung tissues were fixed and paraffin-embedded, and 5-μm sections were stained using hematoxylin and eosin and examined using light microscopy. Scoring of the degree of staining was based on a previously reported metric (35).

### *In vivo* phagocytosis assays.

*IL-6^−/−^* and WT mice with pneumococcal pneumosepsis were administered fluorescein isothiocyanate (FITC)-labeled, heat-killed S. pneumoniae, and BALF cells were collected 4 h later. Cell nuclei were stained with 10 μg/mL DAPI (4′,6-diamidino-2-phenylindole), and fluorescent images were then observed and analyzed using a fluorescence microscope (Nikon ECLIPSE 80i, Japan).

### *Ex vivo* killing assays.

Peritoneal macrophages were infected with 2 × 10^7^ CFU of S. pneumoniae to give a multiplicity of infection (MOI) of 100, and cultured for 1 h to allow phagocytosis. The plates (T0 and T2) were washed with PBS, gentamicin (200 μg/mL) and penicillin (10 μg/mL) were added, and then the plates were incubated for 15 min to kill free and extracellular adherent bacteria. The T0 and T2 samples were lysed at 0 and 1 h, respectively. CFU was determined by plating on blood agar plates as previously described (14).

### Flow cytometry analysis.

Total leukocyte counts in BALF were determined using a hemocytometer. Myeloid cells were quantified by incubating BALF cells with purified anti-mouse CD16/32 (BD Biosciences, USA) and stained with allophycocyanin (APC)-conjugated anti-CD11b (BD), FITC-conjugated anti-Ly-6G (BD), FITC-conjugated anti-CD11c (BioLegend, USA) and phycoerythrin-conjugated anti-F4/80 (BD).

To differentiate early-stage apoptotic cells from late-stage apoptotic and necrotic cells, BALF cells were stained with both FITC Annexin V and PI (BD Biosciences) in accordance with the manufacturer’s instructions. To evaluate necrosis induction in macrophages, cells were stained using PI, 7-AAD (BD), and macrophage-specific antibodies (see above). Subsequently, alveolar macrophages were gated according to their FSC/SSC, F4/80-PE, and CD11c-FITC cell surface expression, and other macrophages were gated according to their FSC/SSC, F4/80-PE, and CD11b-APC cell surface expression, followed by determination of the percentage of PI/7-AAD^+^-resistant alveolar macrophages and recruitment macrophages as previously described (34).

### *In situ* cell death detection.

Detection and quantification of apoptosis in lung tissues and cells were performed on paraffin-embedded tissue sections or in fixed cultured cells using the *In Situ* Cell Death Detection Kit (Roche, Switzerland) according to the manufacturer’s protocol. The fluorescent images were analyzed using a fluorescence microscope (Nikon).

### Immunoblotting.

Cell lysates were separated using 10% SDS-PAGE and transferred onto Immobilon PVF-membranes (Millipore, USA) by electrotransfer. A protein size ladder (Thermo Fisher Scientific, USA) was used for size comparison. The primary antibodies used were as follows: cleaved caspase-3 (Cell Signaling Technology, USA), GSDME (Abcam, UK), caspase-1, GSDMD, IL-1β, IL-18 (all Abcam) and glyceraldehyde-3-phosphate dehydrogenase (ProteinTech Group, USA). Secondary horseradish peroxidase-coupled antibodies (KPL, USA) and Super ECL Plus chemiluminescent detection substrate (US Everbright and Immobilon Western) were used for detection. The protein bands were analyzed using an ECL chemiluminescent detection system (Bio-Rad, CA) and band intensity was quantified using ImageJ software version 1.8.0 (https://imagej.nih.gov/ij/).

### Immunohistochemistry.

Immunohistochemistry was performed using the mouse monoclonal cleaved caspase-3 antibody. The sections were visualized under a light microscope at ×100 magnification.

### IL-6 treatments.

IL-6 was administered to mice intranasally with 10 μg/30 μL mouse recombinant IL-6 protein (Beyotime, China) at the same time the animals were challenged with S. pneumoniae. Cells were treated with exogenous recombinant mouse IL-6 protein (20 ng/mL) at the same time as S. pneumoniae was added.

### *Ex vivo* infection.

Cells were infected with S. pneumoniae D39 at an MOI of 100. For further processing, cells were lysed for Western blotting or fixed for fluorescent staining. Heat inactivation was performed at 60°C for 30 min.

### Statistical analysis.

Data are presented as medians and error bars in all graphs, indicating standard deviation (SD) and representing biological replicates. For survival studies, significance was assessed by log-rank (Mantel-Cox) test. For other data (pneumococcal loads, cytokine and protein levels), statistical significance was determined by the Mann-Whitney U-test (two comparisons) or the Kruskal-Wallis test (multigroup comparisons). All statistical comparisons were performed using Prism 7 (GraphPad Software, USA). The levels of statistical significance were designated as *, *P* < 0.05; **, *P* < 0.01; and ***, *P* < 0.001.
