# Supplementary material for: IL-6 Prevents Lung Macrophage Death and Lung Inflammation Injury by Inhibiting GSDME- and GSDMD-Mediated Pyroptosis during Pneumococcal Pneumosepsis
Source: Microbiol Spectr. 2022 Mar 17;10(2):e02049-21. doi: 10.1128/spectrum.02049-21 (PMC9045248; doi:10.1128/spectrum.02049-21)
Supplement: SUPPLEMENTAL FILE 1 — Supplemental material. Download SPECTRUM02049-21_Supp_1_seq5.pdf, PDF file, 1.2 MB [file spectrum02049-21_supp_1_seq5.pdf]

Figure S1. IL-6 and BMDM enhance antimicrobial and anti-inflammatory ability of mice with pneumococcal pneumosepsis. (A-D) Pneumococcal load of BALF (n=4-6/group), lung (n=4-6/group), spleen (n=3-6/group) and liver (n=4-6/group) of mice when treated with WT BMDM during pneumococcal pneumosepsis. **(E-G)** H&E staining of lung tissues sections (n=5/group), histologic score (n=5/group) and total protein in BALF (n=4-5/group) of mice when treated with WT BMDM during pneumococcal pneumosepsis. Black arrow, inflammatory cell infiltration; blue arrow, epithelial cell shedding; red arrow, bleeding. **(H)** Pneumococcal load of lung (n=5/group) of mice when treated with $IL\text{-}6^{-/-}$ BMDM during pneumococcal pneumosepsis.

Figure S2. BALF cells during pneumococcal pneumosepsis. Total numbers of inflammatory cells, macrophages and neutrophils in BALF from 1 to 3 dpi during pneumococcal pneumosepsis (n=6-11/group).

Figure S3. CXCL-2 and CXCL-5 levels in infected mice. (**A**) CXCL-2/MIP-2 and (**B**) CXCL-5 in lung homogenates of *S. pneumoniae* infected $IL\text{-}6^{-/-}$ and WT mice 72 hpi as measured by ELISA (n=4-6/group).

Figure S4. IL-6 regulates *S. pneumoniae*-induced lung cell death. (A-B) PI^+ cells (n=4-5/group) and early apoptotic cells (n=11/group) in BALF from mice during *S. pn*eumoniae infection at the indicated times. **(C-D)** Lung cell death in mice during *S. pn*eumoniae infection assessed by *in situ* cell death

detection kit and immunohistochemical staining (cleaved caspase-3) (n=3/group).

Figure S5. IL-6 only minimally affects mRNA levels of pyroptosis-related proteins. *In vivo* mRNA levels of the following proteins from lungs of *S. pneumoniae*- infected *IL-6*$^{-/-}$ and WT mice. (**A**) *Caspase-3* (n=3/group), *Gsdme* (n=3/group) and *Il1β* (n=3-6/group) and **(B)** *Caspase-1* (n=3-5/group), *Gsdmd* (n=3- 5/group) and *Il18* (n=3/group) at the indicated times.

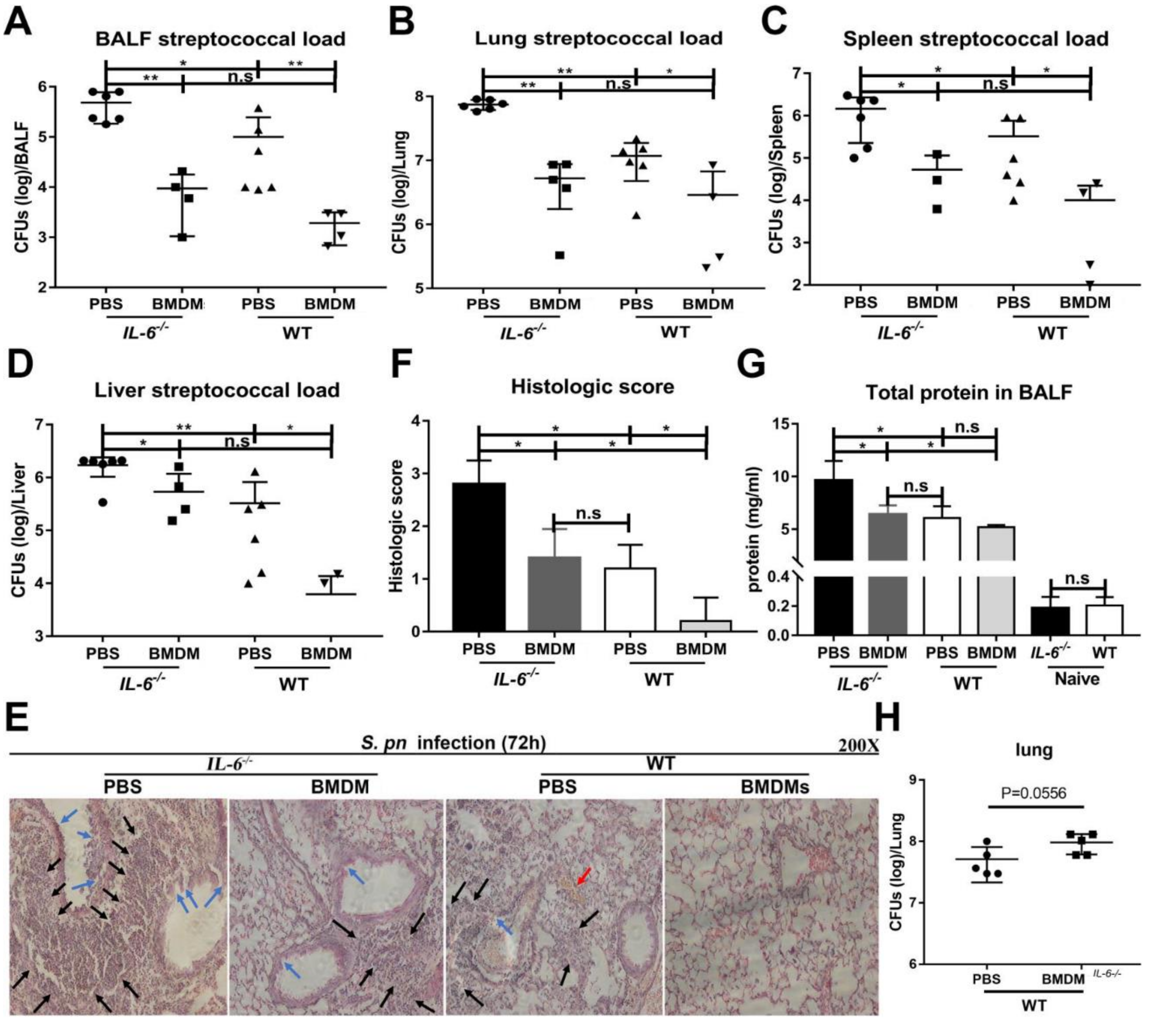
A
BALF streptococcal load
CFUs (log)/BALF
PBS
BMDM
IL-6-/-
WT
n.s
B
Lung streptococcal load
CFUs (log)/Lung
C
Spleen streptococcal load
CFUs (log)/Spleen
D
Liver streptococcal load
CFUs (log)/Liver
F
Histologic score
G
Total protein in BALF
protein (mg/ml)
Naive
E
S. pn infection (72h)
200X
BMDMs
H
lung
P=0.0556

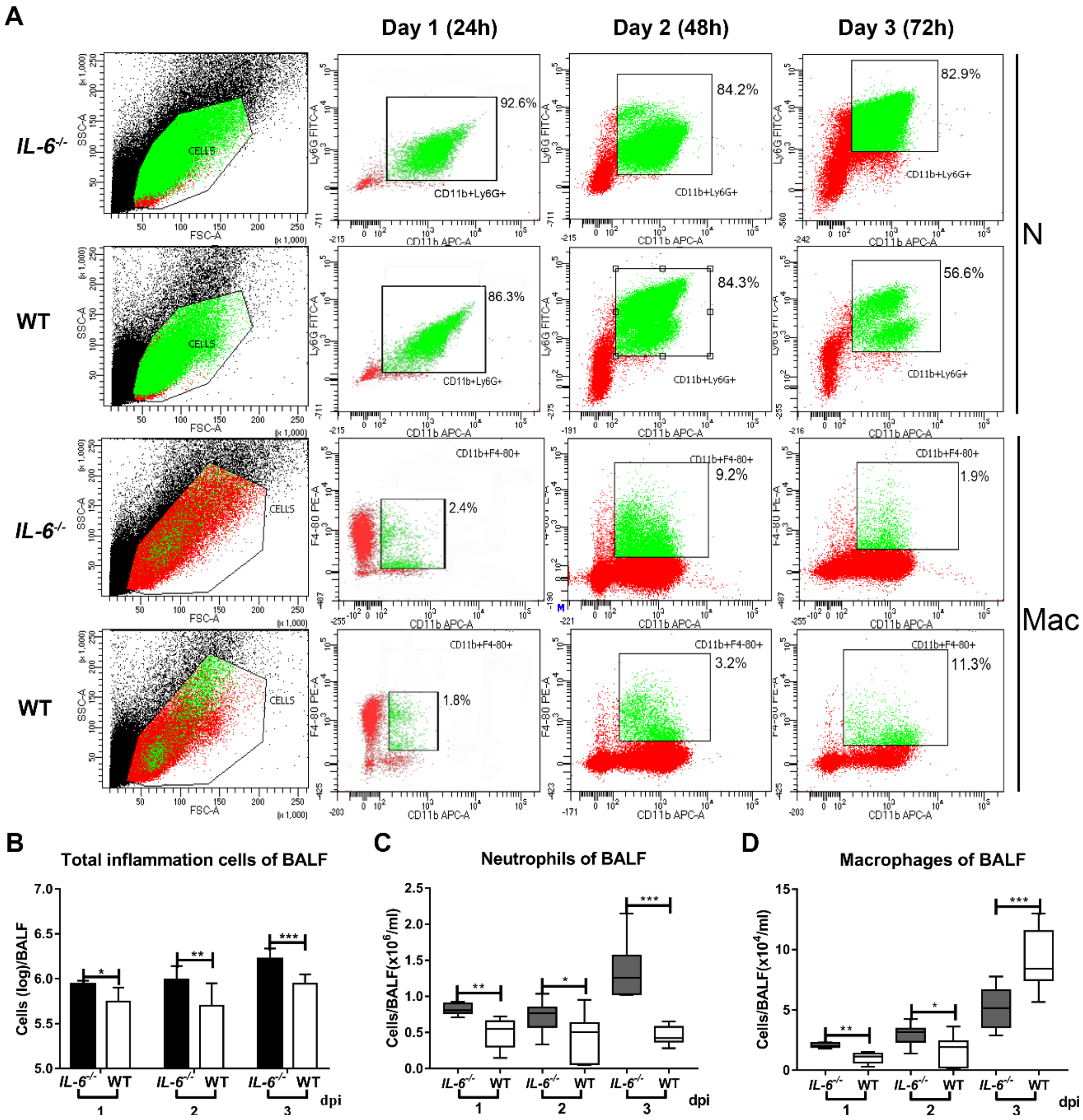
A
Day 1 (24h)
Day 2 (48h)
Day 3 (72h)
IL-6-/-
WT
IL-6-/-
WT
N
Mac
CELLS
SSC-A
FSC-A
Ly6G FITC-A
CD11b APC-A
F4-80 PE-A
CD11b+Ly6G+
CD11b+F4-80+
92.6%
84.2%
82.9%
86.3%
84.3%
56.6%
2.4%
9.2%
1.9%
1.8%
3.2%
11.3%
B
Total inflammation cells of BALF
Cells (log)/BALF
C
Neutrophils of BALF
Cells/BALF(x10^6/ml)
D
Macrophages of BALF
Cells/BALF(x10^4/ml)
IL-6-/-
WT
dpi
1
2
3

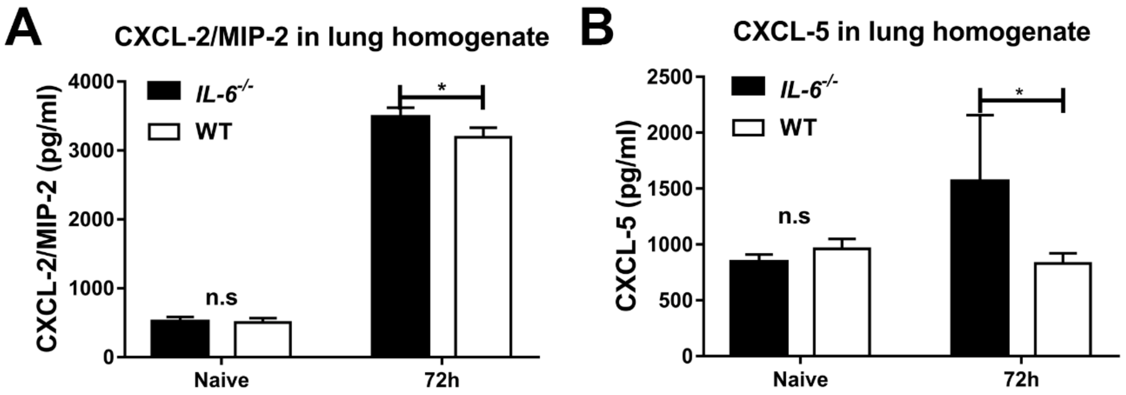
A
CXCL-2/MIP-2 in lung homogenate
CXCL-2/MIP-2 (pg/ml)
4000
3000
2000
1000
0
IL-6-/-
WT
*
n.s
Naive
72h
B
CXCL-5 in lung homogenate
CXCL-5 (pg/ml)
2500
2000
1500
1000
500
0
IL-6-/-
WT
*
n.s
Naive
72h

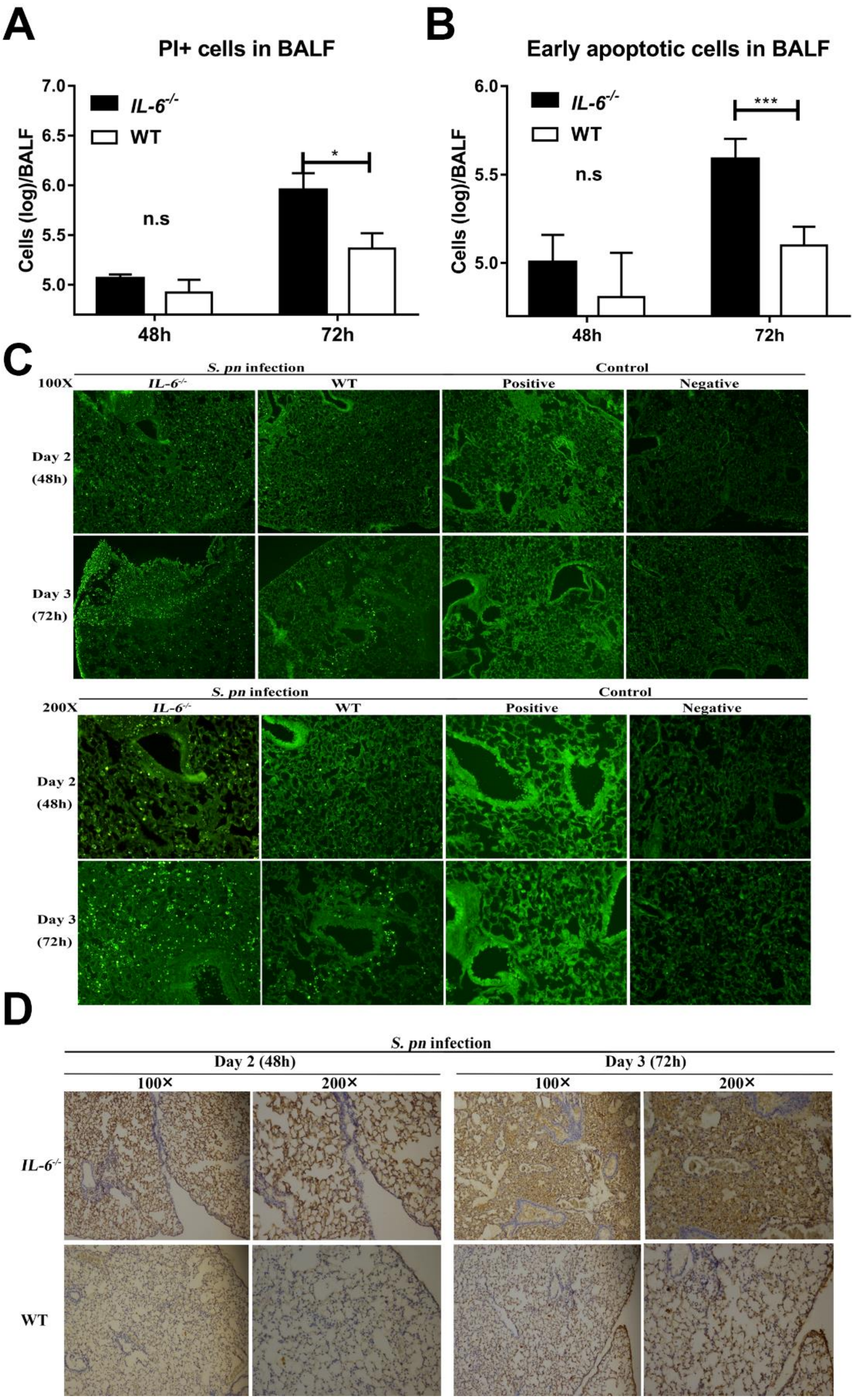
A
PI+ cells in BALF
Cells (log)/BALF
IL-6-/-
WT
n.s
*
48h
72h
B
Early apoptotic cells in BALF
Cells (log)/BALF
IL-6-/-
WT
n.s
***
48h
72h
C
S. pn infection
Control
100X
IL-6-/-
WT
Positive
Negative
Day 2 (48h)
Day 3 (72h)
200X
D
S. pn infection
Day 2 (48h)
Day 3 (72h)
100×
200×
IL-6-/-
WT

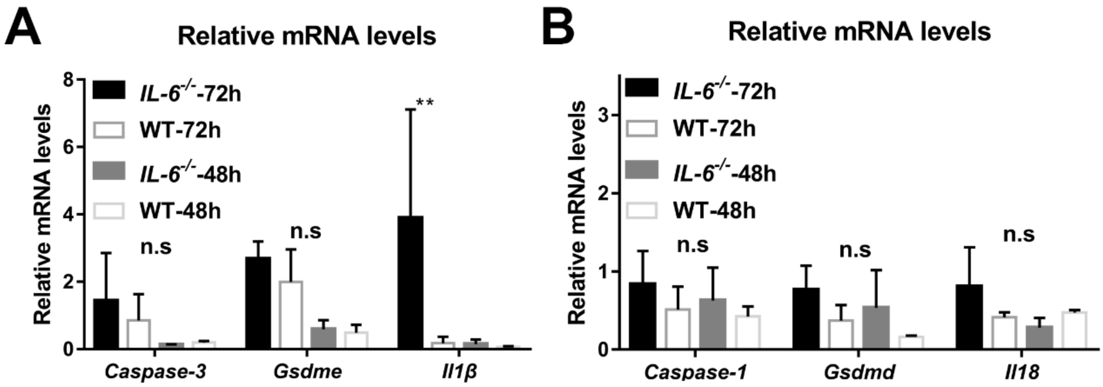
A
Relative mRNA levels
Relative mRNA levels
IL-6-/--72h
WT-72h
IL-6-/--48h
WT-48h
n.s
n.s
**
Caspase-3
Gsdme
Il1β
B
Relative mRNA levels
Relative mRNA levels
IL-6-/--72h
WT-72h
IL-6-/--48h
WT-48h
n.s
n.s
n.s
Caspase-1
Gsdmd
Il18
